# iBitter-HF: A Method for Bitter Peptide Sequence Identification Based on Hybrid Feature Embedding

**DOI:** 10.3390/foods15173016

**Published:** 2026-08-27

**Authors:** Feng Yan, Shicheng Xiang, Yi Tang, Zhengran Kuang, Hengxi Liu, Ximei Luo, Zhibin Lv

**Affiliations:** 1 College of Biomedical Engineering, Sichuan University, Chengdu 610041, China; yanfeng1205@foxmail.com (F.Y.); shichengxiang2004@foxmail.com (S.X.);; 2 Institute of Fundamental and Frontier Sciences, University of Electronic Science and Technology, Chengdu 610106, China; luoximei@uestc.edu.cn

**Keywords:** food protein hydrolysates, peptide bitterness, UniRep, physicochemical descriptors, LightGBM, XGBoost

## Abstract

Bitter peptides are a practical barrier in food-grade protein hydrolysates, fermented products, and peptide-based supplements because they can compromise flavor before nutritional or functional value is realized. Sensory panels and mass-spectrometry-based identification remain reliable, but their throughput is limited for early screening of large peptide pools. Existing predictors usually emphasize either interpretable hand-crafted descriptors or deep sequence representations, whereas these two information sources may be complementary for food-oriented bitter peptide screening. Here, we propose iBitter-HF, a hybrid feature embedding method that integrates seven classes of hand-crafted descriptors with Unified Representation (UniRep) features. Light Gradient Boosting Machine (LGBM)-based feature-importance ranking was used to organize the candidate embeddings, and eXtreme Gradient Boosting (XGB) was used for classification of the selected feature subset. On the public BTP640 benchmark, the finalized 135-feature model achieved 96.9% accuracy on the independent test set. Literature-based comparison indicated competitive performance relative to eight reported bitter peptide predictors, and dimensionality reduction visualization suggested clearer local organization of bitter and non-bitter peptides after feature optimization. These results support iBitter-HF as a computational aid for sequence-level bitter peptide screening and debittering-oriented design of protein hydrolysates.

## 1. Introduction

Food-grade protein hydrolysates are widely used in fermented foods, nutritional formulations and functional peptide products, but their flavor often determines whether these ingredients can be used at practical levels. During enzymatic hydrolysis or processing, proteins release short peptides, and some become bitter because hydrophobic residues are exposed, specific residue patterns are formed, or bitter taste receptors are activated [1,2,3]. Once present, bitter peptides can lower consumer acceptance and force additional debittering, reformulation, or batch-level quality control. Conventional identification relies on separation, mass spectrometry and sensory evaluation [4]. These methods remain the reference for confirming bitterness, but they are not efficient enough for screening hundreds or thousands of candidate peptide sequences in the early design stage. A sequence-based machine-learning model can move part of this decision earlier, before synthesis or isolation, and can help prioritize peptides for food protein utilization and functional product design [5,6].

Progress in machine-learning-based bitter peptide prediction has not followed one straight line. One line of work has focused on feature extraction, where dipeptide propensities, composition profiles, or multi-view descriptors are used to encode sequence differences relevant to taste [7]. iBitter-SCM, for instance, built a scoring-card model from dipeptide propensity scores and reached 84.4% accuracy on the independent test set [8]. A second set of models brought in richer sequence representations. BERT4Bitter, iBitter-Fuse and iBitter-DRLF used BERT, multi-view features and UniRep/BiLSTM representations, respectively, and increased accuracy to 92.2%, 93.0% and 94.4% [9,10,11]. A third line has emphasized the classifier, including random forests, gradient boosting and ensemble learning, to better model nonlinear decision boundaries [12,13,14,15,16]. More recent systems combine representation and optimization; iBitter-GRE and iBitter-Stack both reached 96.1% accuracy [12,13,14,15]. This development indicates that useful predictors need both informative sequence descriptors and algorithms that can exploit them while controlling redundancy, overfitting risk, and interpretability.

Why combine two feature families? Hand-crafted descriptors encode amino acid composition, residue pairs, physicochemical properties, sequence order and substitution tendency [17,18,19]. These descriptors are attractive in food peptide work because hydrophobicity, charge, aromaticity and residue arrangement can be related back to taste-relevant chemistry. The limitation is also obvious: predefined descriptors only see the patterns they are designed to see [20]. UniRep, by contrast, is a protein sequence representation learned with an mLSTM model from large amino acid sequence corpora [21]. It can capture latent context in a peptide sequence, but the resulting dimensions are less directly interpretable. UniRep has already been used for bitter peptide prediction [11]. What has not been tested systematically is whether a ranked and optimized embedding that combines multiple hand-crafted descriptors with the full UniRep representation can improve bitter peptide recognition while retaining some biochemical interpretability.

Accordingly, iBitter-HF was designed to test whether a ranked hybrid feature embedding can improve bitter peptide recognition over either hand-crafted descriptors or UniRep alone. The study compares feature sources under the same BTP640 split, constructs a compact feature subset from the predefined training data, and evaluates it on the independent test set using multiple discrimination metrics. The final Top 135 model achieved 96.9% independent-test accuracy and retained strong MCC, F1 score, auROC, and auPRC values, but the claim is framed as benchmark-level sequence screening rather than sensory bitterness-intensity prediction. By combining dimensionality-reduction visualization, feature-source analysis, and webserver deployment, this work provides a practical computational aid for prioritizing bitter peptide candidates while clarifying the validation boundaries that remain for future food-matrix studies.

## 2. Materials and Methods

**Figure 1:**
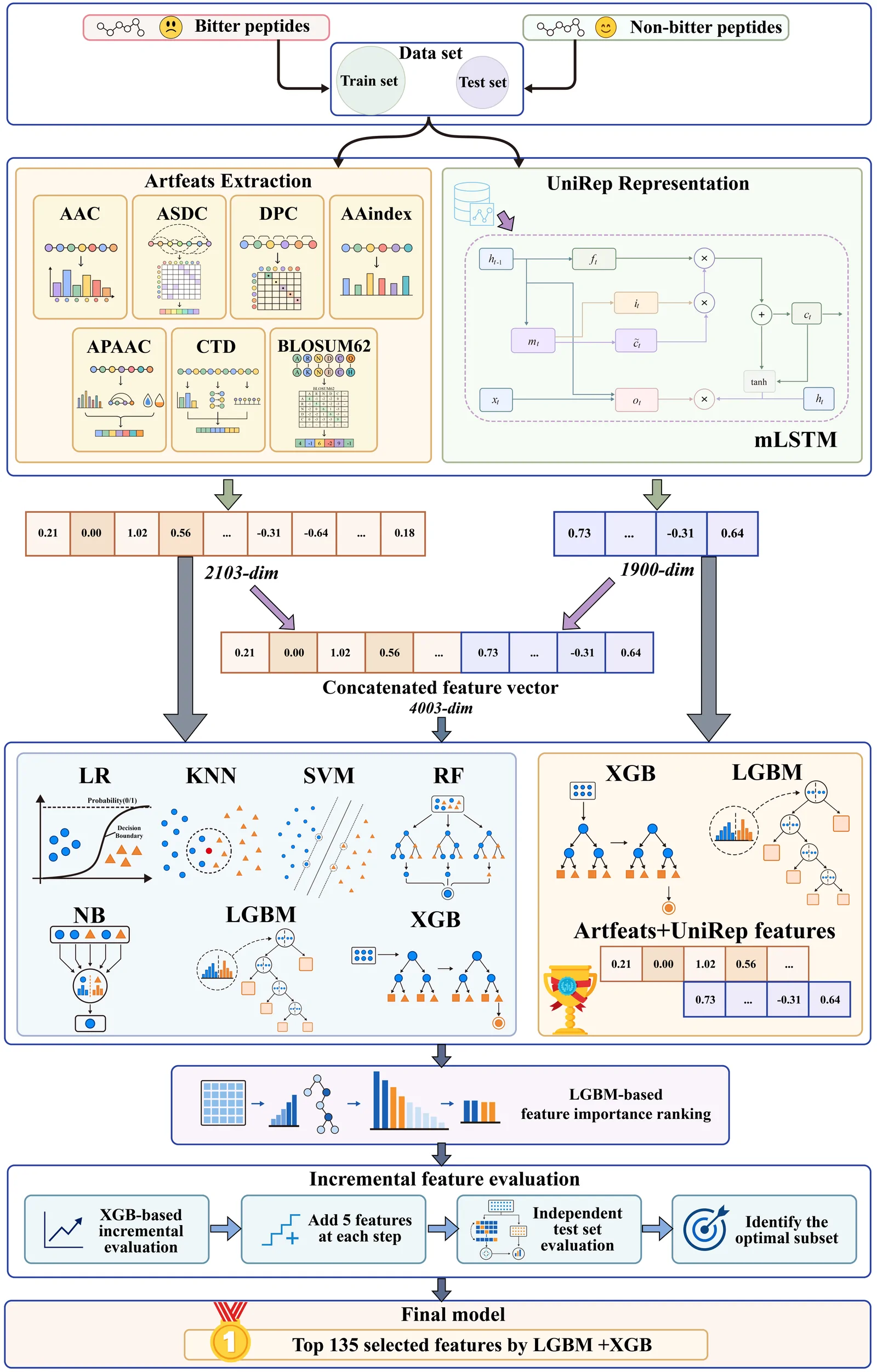
Overall workflow of iBitter-HF. The diagram shows dataset partitioning, feature extraction, construction of the candidate hybrid feature embedding, model comparison, training-set LGBM-based feature ranking, ranked feature-subset evaluation, dimensionality reduction visualization, final model output, and webserver deployment.

The construction of iBitter-HF included dataset preparation, feature extraction, candidate hybrid feature embedding, machine-learning model comparison, training-set LGBM-based feature-importance ranking, ranked feature-subset evaluation, independent-test assessment, dimensionality reduction visualization, and webserver deployment. The overall workflow is shown in Figure 1.

### 2.1. Dataset

This study used the BTP640 benchmark dataset [8,9,10,11]. The dataset was originally constructed for the iBitter-SCM model and was subsequently adopted by studies such as iBitter-DRLF. It has become a commonly used balanced dataset for bitter peptide sequence prediction. BTP640 contains 320 bitter peptides and 320 non-bitter peptides. The training set contained 512 sequences, including 256 bitter peptides and 256 non-bitter peptides, whereas the independent test set contained 128 sequences, with 64 samples in each class. The complete training set was used once for supervised LGBM feature ranking and mean-importance thresholding. Cross-validation was subsequently applied to the fixed selected training matrix; feature selection was not repeated within each fold. Because the selection step preceded cross-validation, these internal summaries may be optimistically biased and are interpreted descriptively. The independent test set was excluded from fitting the selector and defining the threshold. After the feature ranking and threshold had been fixed, the independent test set was used to report benchmark performance and to generate the post hoc ranked-subset sensitivity curve; that curve was not used to determine the Top 135 subset. Exact duplicate checking showed 512 unique training sequences, 128 unique independent-test sequences, and no identical sequence overlap between the two partitions. Because many BTP640 peptides are short, we also performed an exploratory pairwise similarity screen: 76 train-test pairs had global sequence identity ≥0.80 and 11 pairs had global sequence identity ≥0.90, while containment-based motif similarity was more frequent. Therefore, exact duplicates were excluded, but homology-level or motif-level redundancy cannot be fully ruled out and is treated as a limitation. Cross-study comparisons used the independent-test metrics reported in the corresponding publications.

### 2.2. Feature Extraction

#### 2.2.1. Hand-Crafted Feature Extraction

Hand-crafted features convert peptide sequences into numerical descriptors with explicit biochemical meaning. Following established protein and peptide sequence encoding strategies [17,18,22,23], we extracted seven classes of features: amino acid composition (AAC), adaptive skip dipeptide composition (ASDC), dipeptide composition (DPC), amino acid index (AAindex), amphiphilic pseudo-amino acid composition (APAAC), composition-transition-distribution (CTD) and BLOSUM62 (blocks substitution matrix 62). These descriptors produced 2103 dimensions in total. According to the objects they describe, they can be grouped into composition and sequence-pattern descriptors, and physicochemical-property and substitution-matrix descriptors.

(1) Composition and sequence-pattern descriptors.

AAC describes the relative frequency of the 20 standard amino acids in a sequence and can reflect the overall proportion of hydrophobic, aromatic or charged residues in a peptide. DPC captures adjacent dipeptide composition and therefore supplements information on neighboring residue relationships. ASDC further extends DPC by considering interval distances, allowing it to represent combinatorial patterns between non-adjacent residues. Let L denote the peptide length, n_i_ the count of the ith amino acid, and n_ij_ and n_ij_^(d)^ the counts of dipeptide ij at adjacent positions and at interval d, respectively. The corresponding features can be expressed as follows:

(1) $${AAC}_{i}\;=\;\frac{n_{i}}{L}$$

(2) $${DPC}_{ij}=\frac{n_{ij}}{L-1}$$

(3) $${ASDC}_{ij}=\frac{\sum_{d}n_{ij}^{\left(d\right)}}{\sum_{i}\sum_{j}\sum_{d}n_{ij}^{\left(d\right)}}$$

APAAC adds sequence-order correlation factors and amphiphilic information to amino acid composition, thereby describing global correlations generated by residue arrangement along the sequence. In this study, 24 APAAC features were extracted.

(2) Physicochemical-property and substitution-matrix descriptors.

AAindex uses amino acid physicochemical-property indices to describe sequence attributes. For the kth physicochemical property, if residue s_t_ has property value p_k_(s_t_), the sequence-level feature can be written as follows [24]:

(4) $$X_{k}\;=\;\frac{1}{L}\sum_{t}A_{k}(s_{t})$$

CTD first divides the 20 amino acids into three groups according to different physicochemical properties, and then calculates composition, class transition, and distribution positions to describe peptide sequences from the perspective of property distribution. BLOSUM62 represents each residue as a 20-dimensional score vector from the substitution matrix, introducing evolutionary substitution tendencies of amino acids [25,26]. These hand-crafted features are interpretable and easy to relate to biochemical properties, but their expressive capacity is constrained by predefined rules. They therefore need to be complemented by deep representation learning features.

#### 2.2.2. UniRep Deep Representation Learning Feature Extraction

UniRep features were extracted following the processing strategy used by Jiang et al. in a deep representation learning study of bitter peptides [11]. Unified Representation (UniRep) is a deep representation learning method pretrained on protein sequences. The original model uses mLSTM to learn contextual dependencies from large-scale amino acid sequences and encodes input sequences as continuous vectors [21]. For each peptide sequence, the model reads the amino acid residue sequence and outputs a 1900-dimensional representation. This representation does not correspond directly to a single physicochemical property, but captures latent patterns automatically learned from sequence data by the pretrained model.

The key idea of mLSTM is to introduce a multiplicative interaction between the input x_t_ and the previous hidden state h_t−1_, thereby enhancing the model’s ability to represent residue-combination relationships:

(5) $$m_{t}\;=\;(W_{m}x_{t})\;⊙\;(U_{m}h_{t-1})$$

(6) $$c_{t}=f_{t}\;⊙\;c_{t-1}+i_{t}\;⊙\;c_{t}^{\sim }$$

(7) $$h_{t}=o_{t}\;⊙\;tanh(c_{t})$$

where m_t_ is the multiplicative intermediate state; i_t_, f_t_ and o_t_ are the input, forget and output gates, respectively; c_t_ is the cell state; and h_t_ is the hidden state. Compared with hand-crafted features, UniRep can automatically learn higher-order patterns from sequence context, but its interpretability is weaker. It is therefore well suited to be combined with hand-crafted features in a candidate hybrid feature embedding.

#### 2.2.3. Construction of the Hybrid Feature Embedding

The hybrid feature embedding in this study was not a direct use of concatenated features as the final model input. Instead, the 2103-dimensional hand-crafted features and the 1900-dimensional UniRep features were first concatenated to form a 4003-dimensional candidate embedding space. LGBM was fitted once on the complete training-set feature matrix, and its default split-count importance was used to rank the candidate features. Features with importance values greater than the mean importance were retained, and the same fixed indices were then extracted from the independent test matrix. After ranking and screening, the final embedding contained both deep representation learning features and high-contribution hand-crafted features, allowing it to combine contextual expression with physicochemical interpretability.

### 2.3. Machine-Learning Models

Seven classifiers were compared under the same data split and feature-input conditions: logistic regression (LR) [27,28], k-nearest neighbors (KNN) [29], naive Bayes (NB) [30], support vector machine (SVM) [31,32], random forest (RF) [33,34], Light Gradient Boosting Machine (LGBM) and eXtreme Gradient Boosting (XGB). These models cover representative linear, distance-based, probabilistic, kernel-based and tree-ensemble modelling strategies. The basic principles of the candidate algorithms and the main hyperparameters used in this study are provided in Supplementary Information File S1, whereas the main text focuses on XGB, the final classifier used in this study.

XGB is based on gradient-boosted decision trees. It iteratively adds new trees to fit the prediction errors remaining from previous rounds, and uses a second-order approximation of the loss function to evaluate candidate splits [35,36]. Its objective function contains both empirical loss and tree-complexity regularization terms, with penalties on the number of leaves and leaf weights to constrain model complexity. Learning rate, row sampling, and column sampling further reduce overfitting risk. This mechanism enables XGB to capture nonlinear relationships and feature interactions in high-dimensional hybrid features, and it was therefore used for performance evaluation of incremental feature subsets and for final classification.

### 2.4. LGBM-Based Feature-Importance Ranking and Model Optimization

High-dimensional hybrid feature embeddings may contain both useful information and redundant noise, making feature selection necessary. Filter methods select features according to data-derived criteria without repeatedly evaluating candidate subsets with the final classifier; embedded methods estimate feature contribution during model fitting; and wrapper methods compare subsets using classifier performance [36,37]. The present procedure was a supervised, model-based embedded selection step rather than a wrapper search. Following the author-supplied code, LGBM was fitted once on the complete predefined training-set feature matrix. Because importance_type was not specified, feature_importances_ represented the default split importance, namely the number of times each feature was used for a tree split. The arithmetic mean of the split-count importance values across all candidate features was used as the threshold. Features strictly above this mean were retained and ordered from highest to lowest importance, producing the fixed Top 135 subset. The same feature indices were then extracted from the independent test matrix. The independent test set was not used to fit LGBM, calculate importance values, or define the threshold. XGB was used after this fixed selection step for classification. Feature selection was not repeated within individual cross-validation folds, and nested feature selection was not implemented. Therefore, internal cross-validation performed after global selection may be optimistically biased and is treated as descriptive.

LGBM belongs to the gradient-boosting tree framework. It iteratively builds decision trees to fit the negative gradient of the current loss function and combines multiple weak learners into a nonlinear classifier [35,36]. Previous studies have shown that this approach can provide predictive performance and computational efficiency in high-dimensional biological data [36,38,39,40,41]. In the supplied implementation, LGBM feature_importances_ used the default split importance. Thus, a feature received a larger importance value when it was selected more often for node splitting across the fitted trees; the value was not a gain-based estimate of reduction in the objective function. The LGBM selection model used num_leaves = 32, n_estimators = 888, max_depth = 12, learning_rate = 0.16, min_child_samples = 50 and random_state = 2020. The final Top 135 XGB classifier was trained on standardized selected features using a binary logistic objective, with n_estimators = 1150, max_depth = 5, learning_rate = 0.0349, subsample = 0.9873, colsample_bytree = 0.9674, reg_alpha = 2.4958, reg_lambda = 0.0274 and random_state = 42; additional parameter details are listed in Supplementary Information File S1.

### 2.5. Evaluation Metrics

**Figure 2:**
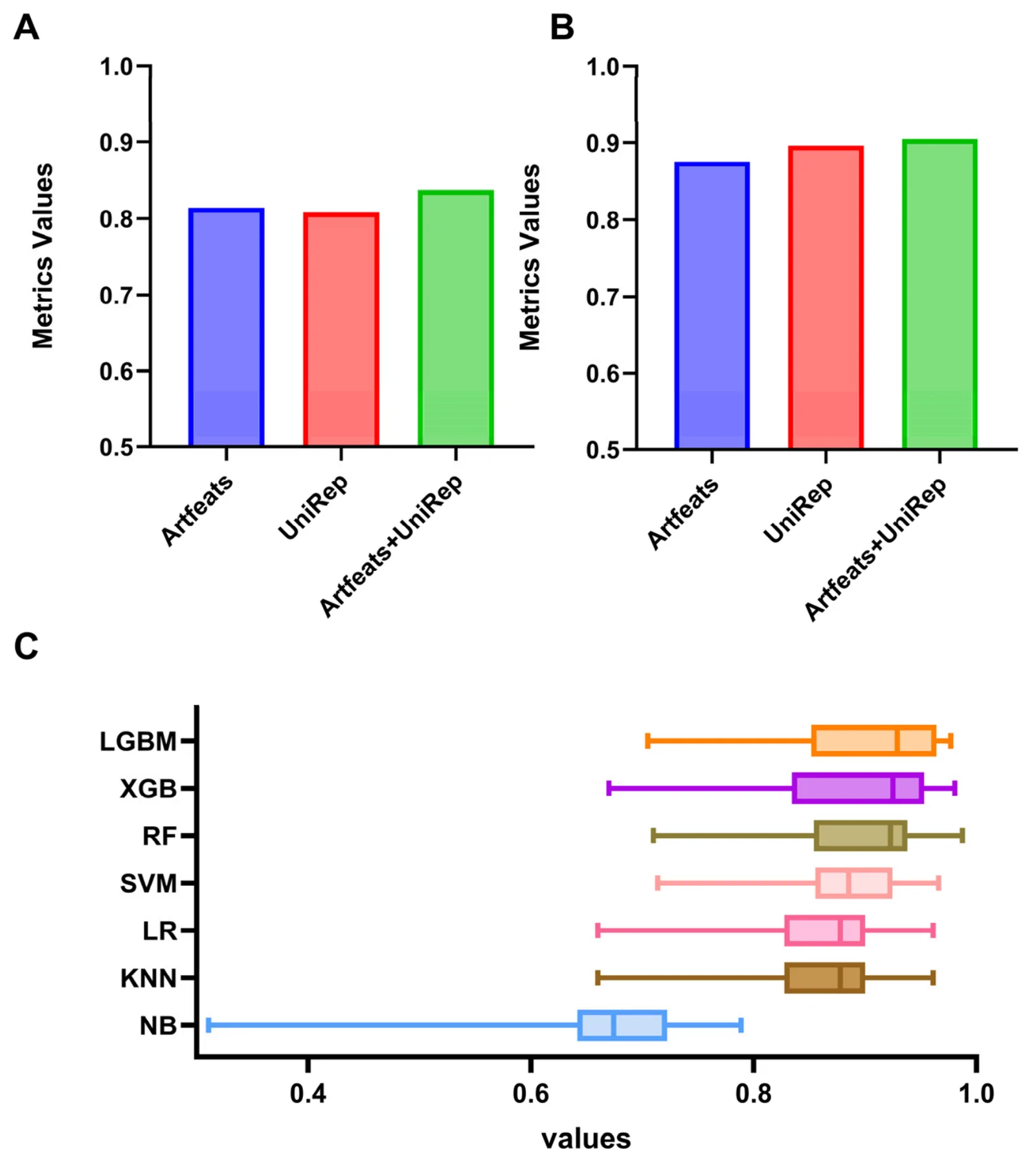
Overall performance comparison of feature representations and machine-learning models. (**A**) Descriptive summary of composite metrics for Artfeats, UniRep and Artfeats + UniRep in the internal validation results. (**B**) Descriptive summary of composite metrics for the three feature representations on the independent test set. (**C**) Distribution of the evaluation metrics for seven candidate classifiers using the Artfeats + UniRep representation. In (**A**,**B**), the bars indicate medians of the grouped performance values obtained under the seven candidate classifiers; no inferential intervals are shown. In (**C**), the central line indicates the median, the box indicates the interquartile range, and the whiskers extend from the minimum to the maximum.

Model performance was evaluated using accuracy (ACC), sensitivity (Sn), specificity (Sp), precision, F1 score, Matthews correlation coefficient (MCC), area under the receiver operating characteristic curve (auROC), and area under the precision-recall curve (auPRC). Let TP, TN, FP and FN denote true positives, true negatives, false positives and false negatives, respectively [42]. For Figure 2A,B, the grouped performance values were entered into GraphPad Prism 11.0.1 and summarized descriptively; the bars show medians across the values obtained under the seven candidate classifiers. The graphical error bars in the originally submitted figure were not generated from a prespecified statistical uncertainty calculation and have therefore been removed. For Figure 2C, the central line indicates the median, the box indicates the interquartile range, and the whiskers extend from the minimum to the maximum. These graphical summaries were not treated as formal hypothesis tests. The main metrics were calculated as follows:

(8) $$ACC\;=\;\frac{TP\;+\;TN}{TP\;+\;TN\;+\;FP\;+\;FN}$$

(9) $$Sn=\frac{TP}{TP+FN},\;\;Sp=\frac{TN}{TN+FP}$$

(10) $$Precision=\frac{TP}{TP+FP}$$

(11) $$F1=\frac{2\times Precision\times Sn}{Precision+Sn}$$

(12) $$MCC=\frac{TP\times TN-FP\times FN}{\sqrt{\left(TP+FP)(TP+FN)(TN+FP)(TN+FN\right)}}$$

ACC reflects the overall proportion of correctly classified samples. Sn measures the proportion of bitter peptides that are correctly identified, whereas Sp measures the proportion of non-bitter peptides that are correctly identified. MCC jointly accounts for all four outcomes in the confusion matrix and is therefore better suited to evaluating balanced binary discrimination. auROC and auPRC reflect the model’s ranking ability across different thresholds and range from 0 to 1 [43,44,45]. Values closer to 1 indicate stronger discrimination, whereas an auROC close to 0.5 indicates performance close to random classification.

### 2.6. t-SNE Visualization Procedure

To support visual inspection of sample distributions under different feature representations, we used t-distributed stochastic neighbor embedding (t-SNE) for two-dimensional visualization [46]. This method first converts distances between samples in the high-dimensional space into conditional similarity probabilities using a Gaussian kernel, with perplexity controlling the effective neighborhood size. It then constructs corresponding probabilities in the low-dimensional space using a heavy-tailed Student t distribution to mitigate crowding among distant samples. The optimization minimizes the Kullback-Leibler divergence between the high- and low-dimensional probability distributions, preserving local neighborhood relationships as much as possible. According to the source-script information for the polished t-SNE visualization panels, all four plots used direct feature input rather than PCA100 pre-reduction, standard scaling, n_components = 2, max_iter = 2000, PCA initialization, and random_state = 42. Panel-specific settings were as follows: Artfeats, Euclidean metric, perplexity = 12 and learning_rate = 185; UniRep, Manhattan metric, perplexity = 5 and learning_rate = 100; candidate hybrid feature embedding, Euclidean metric, perplexity = 14 and learning_rate = 190; and Top 135 selected features, Euclidean metric, perplexity = 14 and learning_rate = 190. t-SNE was used only as qualitative visualization and was not used for model training, feature ranking, or final evaluation.

### 2.7. Webserver Implementation and Usage

To facilitate practical use, an online iBitter-HF webserver was deployed at https://iservers.aibiochem.net/iBitterHF (accessed on 14 August 2026). The webserver interface provides two input modes: FASTA-formatted text input and file upload. The text-input example contains multiple FASTA records, indicating support for batch FASTA-style submission. According to the interface, submitted sequences are parsed into a sequence DataFrame and passed to the prediction function, which returns a binary label and a probability value: label 1 denotes a bitter peptide and label 0 denotes a non-bitter peptide, with probability reported in percent. The interface also states a maximum of 100 sequences per submission, and excess records will be truncated. No explicit residue-length cutoff is displayed on the interface; because the BTP640 benchmark peptides ranged from 2 to 39 amino acids in length, predictions for sequences far outside this range should be interpreted cautiously and followed by experimental validation when used in food applications. No quantitative webserver-throughput benchmark is reported here; consequently, we do not make a quantitative claim about prediction throughput.

## 3. Results and Discussion

### 3.1. Effect of Feature Hybridization on Bitter Peptide Identification

To evaluate the effect of hybrid features on bitter peptide identification, we constructed hand-crafted features (Artfeats), 1900-dimensional UniRep features, and a candidate hybrid feature embedding (Artfeats + UniRep). Seven classifiers were then trained on each representation under the same predefined data split. This direct feature-source comparison served as a representation-level ablation analysis, because the two single-source inputs and their hybrid input were evaluated separately before feature ranking. The median across multiple evaluation metrics was used to summarize overall performance and reduce the influence of extreme values (Figure 2A,B). On the independent test set, Artfeats + UniRep reached a composite metric median of 0.9219, higher than Artfeats (0.9097) and UniRep (0.8950). Its median accuracy was 89.8%, representing increases of 3.90 and 0.78 percentage points over the two single representations, respectively. These results indicate that the compositional and physicochemical information provided by hand-crafted features complements the sequence context encoded by UniRep, giving the hybrid feature embedding more stable overall identification ability.

### 3.2. Comparison of Machine-Learning Models Under Hybrid Feature Embedding

After confirming that hybrid features improved the overall representation, we compared the ability of seven classifiers to use this high-dimensional input (Figure 2C). LGBM and XGB achieved multi-metric means of 0.9549 and 0.9536, respectively, higher than RF (0.9403) and SVM (0.9045), with medians of 0.9604 and 0.9531. Both boosting-tree models achieved an accuracy of 95.3% and an MCC of approximately 0.906. XGB reached an auPRC of 0.9806, whereas LGBM reached an Sp of 0.9688. By contrast, NB had a mean value of only 0.6726 and an MCC as low as 0.3930, indicating that the conditional-independence assumption is inadequate for describing relationships among multi-source features. Considering the mean, median, and extreme metric values together, tree-ensemble models were better suited to capturing nonlinear patterns in the hybrid features. Therefore, LGBM was used for feature ranking and XGB for incremental evaluation.

### 3.3. Model Optimization Based on Feature-Importance Ranking

**Figure 3:**
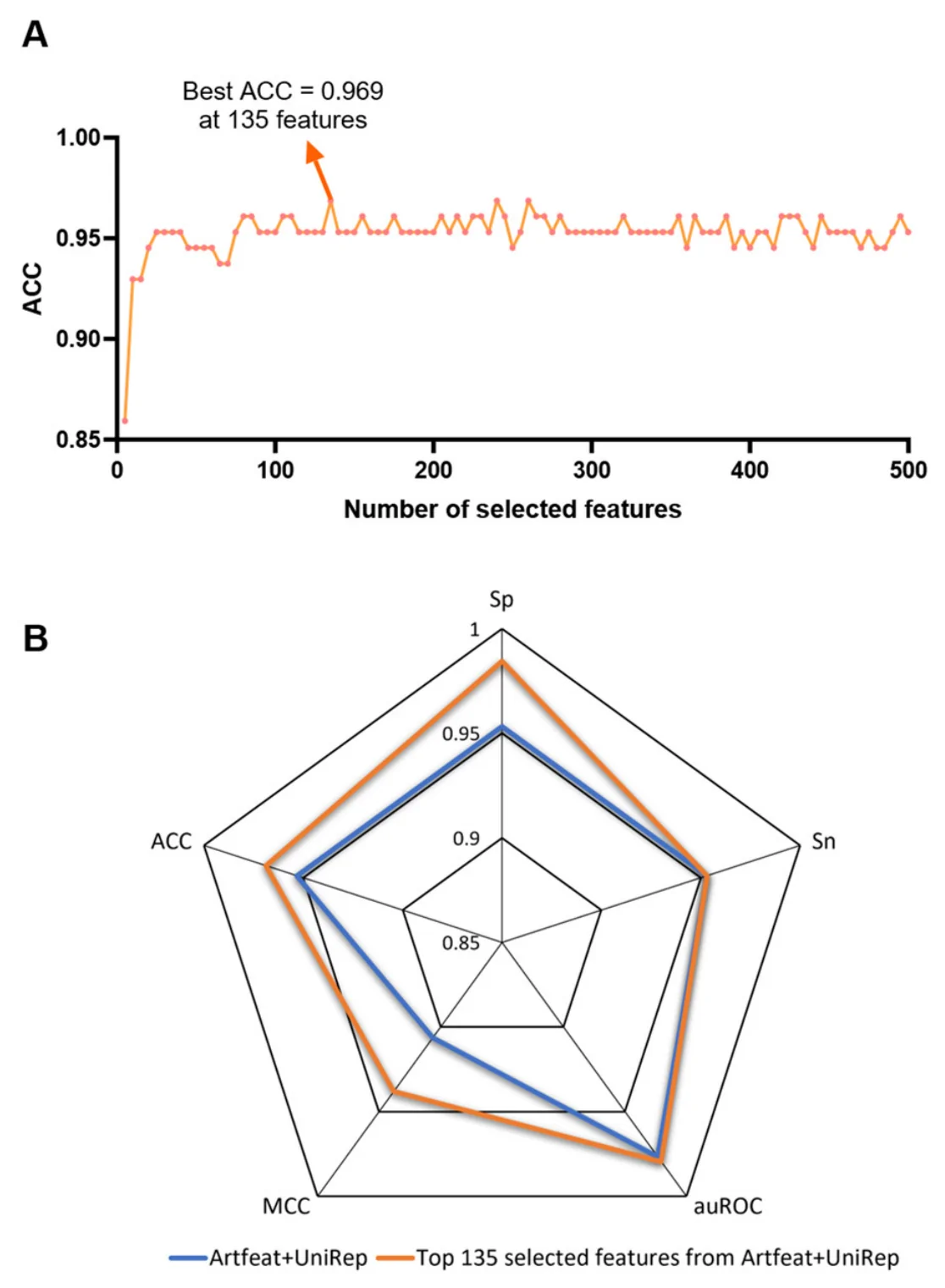
LGBM-based feature-importance ranking and feature-subset analysis. (**A**) Post hoc independent-test accuracy trend for ranked feature subsets after the LGBM ranking had been calculated from the complete training set. The Top 135 subset was determined by retaining training-set features with split-count importance values higher than the mean importance, not by maximizing the curve. Because the independent test set was repeatedly evaluated to draw this descriptive curve, panel A is not treated as an independent estimate of generalization. (**B**) Main evaluation metrics of the original candidate hybrid feature embedding and the fixed Top 135 model on the independent test set.

The candidate hybrid feature embedding contained 4003 variables, combining rich information with potential redundant noise. LGBM was fitted once on the complete training-set feature matrix, and features with default split-count importance greater than the mean importance were retained. This predefined rule produced a fixed Top 135 subset containing 93 UniRep features and 42 hand-crafted descriptors, including 27 AAindex, 14 CTD, and 1 ASDC descriptor. After the rule and indices had been fixed, the Top 135 model achieved ACC = 0.96875, MCC = 0.93796, Sn = 0.95313, Sp = 0.98438, precision = 0.98387, F1 score = 0.96825, auROC = 0.97949, and auPRC = 0.98278 on the independent test set. Figure 3A presents a post hoc sensitivity curve obtained by applying ranked feature subsets to the independent test set. It was not used to choose the mean-importance threshold or the Top 135 feature number and should not be interpreted as a series of independent generalization estimates. Compared with the original candidate embedding, the fixed Top 135 subset increased ACC from 0.95313 to 0.96875, MCC from 0.90625 to 0.93796, Sp from 0.95313 to 0.98438, and auROC from 0.97656 to 0.97949, while Sn remained 0.95313. The selected feature composition suggests that deep contextual information made the major contribution, whereas physicochemical-property and residue-combination descriptors provided complementary interpretable sequence information.

### 3.4. Dimensionality Reduction Visualization of the Feature Space

**Figure 4:**
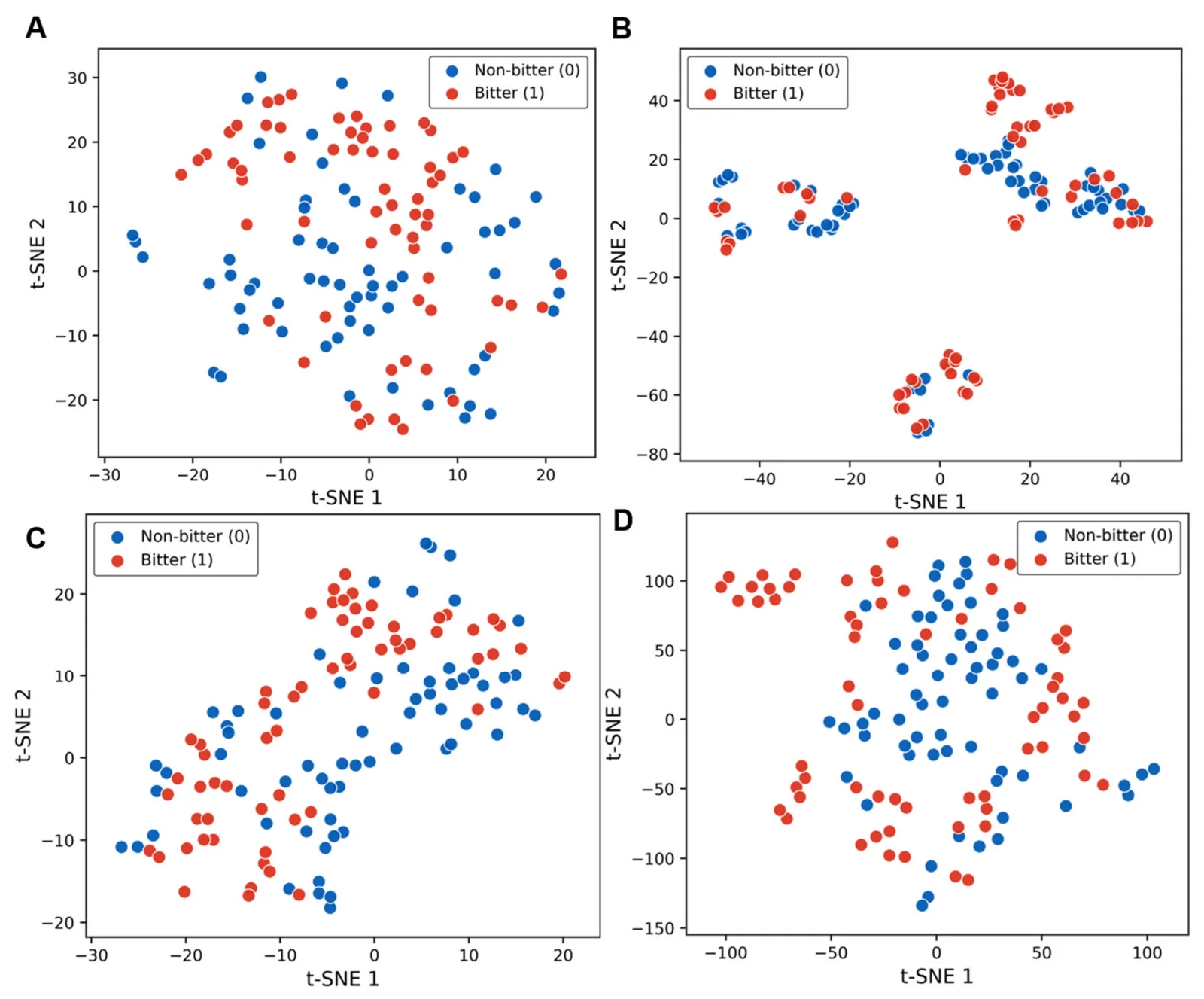
Two-dimensional visualization of different feature representations on the independent test set. (**A**) Hand-crafted features. (**B**) UniRep features. (**C**) Candidate hybrid feature embedding. (**D**) Top 135 features after LGBM-based ranking and feature-subset optimization. t-SNE was used only for qualitative visualization of local sample organization; detailed panel-specific parameters are described in Section 2.

In addition to quantitative metrics, feature-space distributions can provide an intuitive view of differences among representations. Figure 4 compares the two-dimensional distributions of hand-crafted features, UniRep features, the candidate hybrid feature embedding, and the Top 135 features on the independent test set. Bitter and non-bitter peptide samples still showed substantial overlap under single feature representations. The candidate hybrid feature embedding produced a more structured sample distribution, suggesting that combining the two information sources improved feature-space organization. After LGBM ranking and feature-subset optimization, the Top 135 features showed clearer local separation in the low-dimensional visualization. This result is consistent with the performance improvements described above, but it should be interpreted as qualitative support rather than quantitative evidence of generalization.

### 3.5. Comparison with Existing Bitter Peptide Predictors

**Figure 5:**
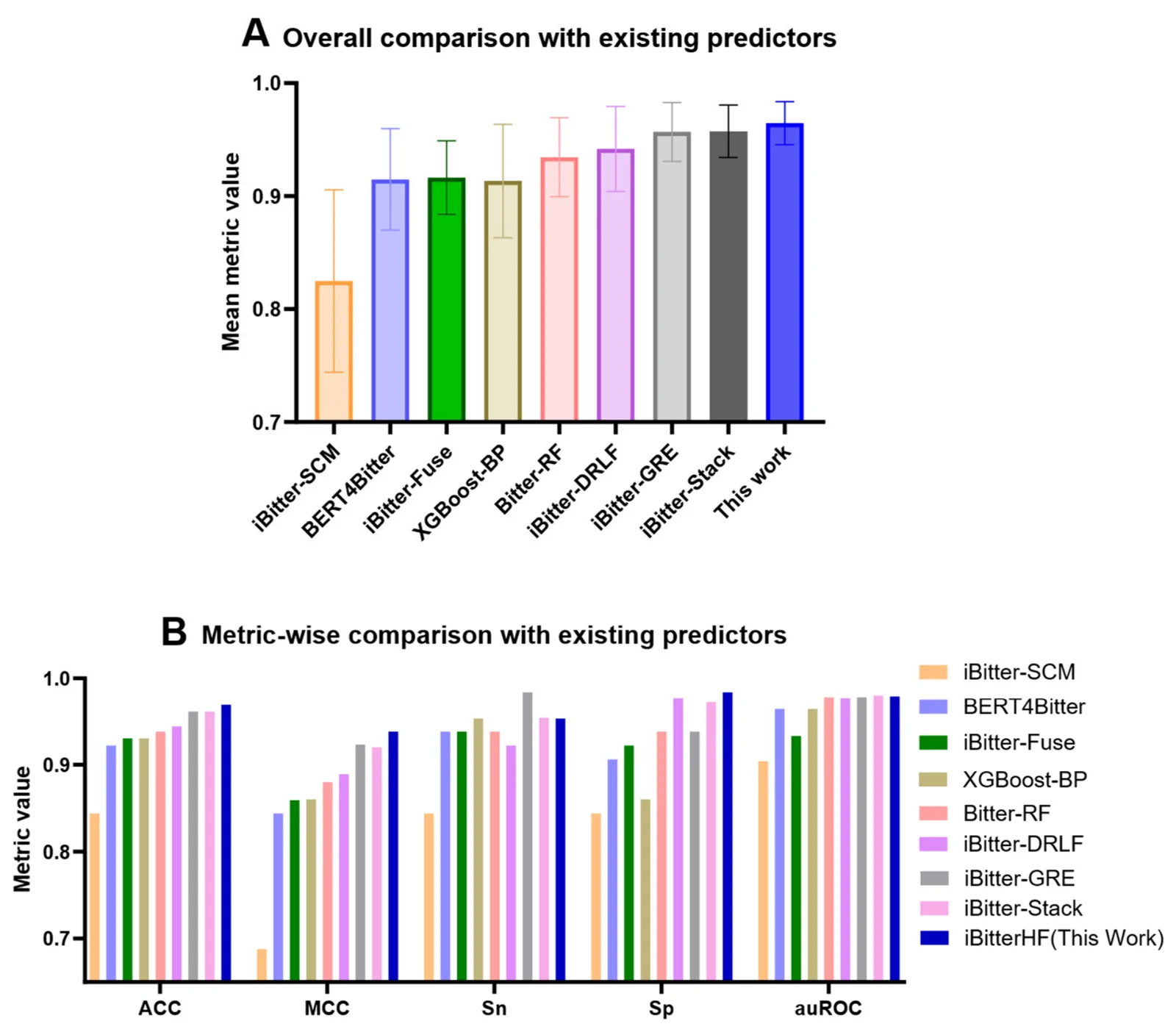
Performance comparison between iBitter-HF and existing bitter peptide predictors. In the plots, the proposed method is labeled as iBitterHF (this work). (**A**) Mean values of five metrics across different models; error bars indicate standard deviations across the five metrics. (**B**) Model-specific results for ACC, MCC, Sn, Sp and auROC. The data for previously published predictors were collected from the reported independent-test metrics, re-organized and plotted by the authors. This figure does not reproduce or adapt any published figure, table or graphical layout.

To evaluate the relative position of iBitter-HF on a public benchmark, we compared it with iBitter-SCM, BERT4Bitter, iBitter-Fuse, iBitter-DRLF, Bitter-RF, iBitter-GRE, iBitter-Stack and XGBoost-BP, using the independent-test results reported in the corresponding studies [8,9,10,11,12,13,14,15]. In Figure 5, the proposed method is labeled as iBitterHF (this work) to distinguish it from previously published predictors. Figure 5A shows that its mean value across five metrics was 0.9646, placing it among the strongest reported models on the BTP640 benchmark; the error bars in this panel indicate standard deviations (SD) across the five metrics, as specified in the original figure annotation. Figure 5B shows that iBitter-HF reached 96.9% ACC and 0.984 Sp, indicating high overall correctness and reliable non-bitter peptide exclusion on this dataset. iBitter-GRE showed higher Sn, and iBitter-Stack had an auROC close to that of iBitter-HF, suggesting that individual predictors emphasize different aspects of discrimination. Because these values were collected from published articles rather than regenerated under a fully unified software environment, this comparison should be interpreted as a descriptive benchmark comparison rather than a formal statistical demonstration of superiority. Overall, the comparison supports iBitter-HF as a competitive screening model when both accuracy and negative-sample exclusion are required.

Several limitations should be noted. First, all experiments were based on the public BTP640 benchmark; although exact duplicate checking found no identical sequence overlap between the training and independent test partitions, an exploratory pairwise similarity screen detected globally high-identity train-test pairs and frequent containment-based motif overlaps, which is expected in part because many peptides in this benchmark are very short. Therefore, possible sequence-similarity redundancy cannot be fully excluded and may influence benchmark performance. Evaluation on additional experimentally characterized food-derived peptide datasets would further test generalization. Second, supervised LGBM feature selection was performed once on the complete training set before cross-validation rather than being repeated within each fold. The resulting internal cross-validation summaries may therefore be optimistically biased and are used descriptively. Although the independent test set did not define the selection threshold, it was repeatedly evaluated in the post hoc Figure 3A sensitivity curve; this panel is consequently not treated as an independent generalization estimate. Third, the comparison with previous predictors was based on literature-reported metrics, so differences in preprocessing, software versions, hyperparameter optimization, and random initialization may affect direct comparability. Fourth, iBitter-HF predicts bitter/non-bitter sequence labels and should not be interpreted as a quantitative predictor of bitterness intensity in real food matrices without sensory or peptidomics validation. Finally, no quantitative webserver-throughput benchmark is reported in the present study. Future work should use fold-wise or nested feature selection, external food-derived peptide datasets, stricter sequence-redundancy control, experimental bitterness measurements, and open-source workflow deposition to further evaluate model generalization and reproducibility.

## 4. Conclusions

This study presents iBitter-HF, a method for bitter peptide sequence identification based on hybrid feature embedding. The method integrates hand-crafted features and UniRep deep representation learning features into a candidate embedding space, and obtains a compact Top 135 feature subset through training-set LGBM feature-importance ranking with a mean-importance threshold. The results showed that the hybrid feature embedding outperformed single feature representations, and that the screened compact feature subset improved model accuracy and non-bitter peptide exclusion on the BTP640 benchmark. In comparison with existing bitter peptide predictors, iBitter-HF achieved 96.9% independent-test accuracy and showed competitive overall performance in the curated benchmark comparison. Dimensionality reduction visualization and feature-source analysis suggested that complementarity between deep contextual information and hand-crafted physicochemical descriptors was an important reason for the performance gain. The method can provide computational support for rapid sequence-level bitter peptide screening, debittering design of protein hydrolysates, and development of functional peptide products, while further validation with externally experimentally characterized food-derived peptides remains an important direction for future work.

## Data Availability

The benchmark dataset analyzed in this study is publicly available from the iBitter dataset repository at https://github.com/Shoombuatong/Dataset-Code/tree/master/iBitter (accessed on 14 August 2026). The training and independent test FASTA files used in this study have been provided during revision for transparency. Processed feature matrices and code are available from the corresponding author upon reasonable request.

## Supplementary Materials

The following supporting information can be downloaded at: https://www.mdpi.com/article/10.3390/foods15173016/s1, File S1. Additional methodological and reproducibility details.
