# Supplementary material for: iBitter-HF: A Method for Bitter Peptide Sequence Identification Based on Hybrid Feature Embedding
Source: Foods. 2026 Aug 27;15(17):3016. doi: 10.3390/foods15173016 (PMC13565165; doi:10.3390/foods15173016)
Supplement: Supplementary file 1 [file foods-15-03016-s001.zip › foods-4457497-supplementary.pdf]

Supplementary Information

File S1. Additional methodological and reproducibility details

This file provides details requested during peer review, including candidate machine-learning algorithms, feature ranking, verified model parameters, t-SNE parameters, web server usage notes, data audit notes, and the preparation of Figure 2.

S1. Dataset and feature matrices

The BTP640 benchmark contains 640 peptide sequences, including 320 bitter and 320 non-bitter peptides. The predefined training set contained 512 sequences, with 256 samples in each class, and the independent test set contained 128 sequences, with 64 samples in each class.

The hand-crafted feature matrix contained 2103 descriptors; the UniRep matrix contained 1900 descriptors; and the candidate hybrid feature embedding contained 4003 dimensions. The selected Top 135 matrix contained 135 feature columns plus sequence/sample identifiers and labels. No missing values were detected in the feature matrices used for modelling.

Exact duplicate checking of the FASTA files showed 512 unique training sequences, 128 unique independent-test sequences, and no exact sequence overlap between the two partitions. Because the benchmark contains many short peptides, we also performed an exploratory pairwise train-test similarity screen using global sequence identity and containment-based motif similarity. The screen identified 76 train-test pairs with global identity >= 0.80 and 11 pairs with global identity >= 0.90. Containment-based motif overlap was more frequent, as expected for very short peptide sequences. These results support the absence of exact duplicates but do not exclude homology-level or motif-level redundancy; this boundary is stated as a limitation in the revised manuscript.

S2. Candidate machine-learning algorithms

Logistic regression (LR): a linear probabilistic classifier used as a simple baseline.

k-nearest neighbours (KNN): a distance-based classifier that assigns labels according to nearby samples.

Naive Bayes (NB): a probabilistic classifier based on conditional-independence assumptions among features.

Support vector machine (SVM): a margin-based classifier that can model nonlinear boundaries through kernel functions.

Random forest (RF): an ensemble of decision trees trained with bootstrap aggregation and random feature selection.

Light Gradient Boosting Machine (LGBM): a gradient-boosting decision-tree model used for feature-importance ranking.

eXtreme Gradient Boosting (XGB): a regularized gradient-boosting decision-tree model used for ranked subset evaluation and final classification.

S3. LGBM feature ranking and XGB model parameters

LGBM-based feature selection was performed once on the complete predefined training-set feature matrix. The LGBM model was fitted using all training features and labels, and feature_importances_ was extracted from the fitted model. Because importance_type was not specified, the default split importance was used; each value records how often a feature was used for a node split across the fitted trees. The arithmetic mean of all candidate split-count importance values was used as the threshold. Features strictly above this mean were retained and ordered from

highest to lowest importance. This rule produced the fixed Top 135 subset. Cross-validation was subsequently applied to this fixed selected training matrix. Feature selection was not repeated within individual folds, and nested feature selection was not implemented; consequently, internal cross-validation summaries may be optimistically biased and are interpreted descriptively. The independent test set was not used to fit LGBM, calculate importance values or define the threshold. After the threshold and selected indices had been fixed, the same indices were extracted from the independent test matrix for benchmark evaluation. Figure 3A additionally reports a post hoc sensitivity curve from repeated independent-test evaluation of ranked subsets; this curve was not used to select the Top 135 subset and is not treated as an independent generalization estimate.

LGBM ranking parameters: num_leaves = 32; n_estimators = 888; max_depth = 12; learning_rate = 0.16; min_child_samples = 50; random_state = 2020; n_jobs = 8.

Top 135 XGB classifier parameters verified from the supplied trained-model file: objective = binary:logistic; n_estimators = 1150; max_depth = 5; learning_rate = 0.0349031698; subsample = 0.9873047378; colsample_bytree = 0.9674334223; reg_alpha = 2.4958342270; reg_lambda = 0.0274027079; random_state = 42. The final classifier was trained on standardized selected features; the supplied StandardScaler recorded 135 input features with mean centering and standard-deviation scaling enabled. The serialized model also recorded tree_method = hist, device = cuda, and predictor = gpu_predictor.

Independent-test metrics for the Top 135 model: ACC = 0.96875; MCC = 0.93796; Sn = 0.95313; Sp = 0.98438; precision = 0.98387; F1 score = 0.96825; auROC = 0.97949; auPRC = 0.98278. Relative to the original candidate hybrid embedding, the Top 135 subset increased ACC from 0.95313 to 0.96875, MCC from 0.90625 to 0.93796, Sp from 0.95313 to 0.98438, and auROC from 0.97656 to 0.97949, while Sn remained 0.95313.

S4. Feature-source composition of the Top 135 subset

The Top 135 feature subset contained 93 UniRep features and 42 hand-crafted descriptors. The hand-crafted descriptors included 27 AAindex features, 14 CTD features, and 1 ASDC descriptor. This composition indicates that UniRep contributed most selected dimensions, whereas physicochemical-property and residue-combination descriptors provided interpretable complementary information.

S5. t-SNE visualization settings

Figure 4 was generated using t-SNE on direct feature inputs rather than PCA100-pre-reduced inputs. All panels used standard scaling, n_components = 2, max_iter = 2000, PCA initialization, and random_state = 42. Panel-specific settings were as follows: Figure 4A (Artfeats), Euclidean metric, perplexity = 12 and learning_rate = 185; Figure 4B (UniRep), Manhattan metric, perplexity = 5 and learning_rate = 100; Figure 4C (candidate hybrid feature embedding), Euclidean metric, perplexity = 14 and learning_rate = 190; Figure 4D (Top 135 selected features), Euclidean metric, perplexity = 14 and learning_rate = 190. These plots were used only for qualitative visualization of local feature-space organization and were not used for model training, feature ranking or final evaluation.

S6. Webserver usage notes

The iBitter-HF webserver is available at https://iservers.aibiochem.net/iBitterHF. The current webserver interface provides two input modes: FASTA-formatted text input and file upload. The example text input contains multiple FASTA records, and the interface states that submitted records are parsed into a sequence DataFrame and passed to the prediction function, which returns a binary label and a probability value: label 1 denotes bitter peptide, label 0 denotes non-bitter peptide, and probability is reported in percent. The interface states a maximum of 100 sequences per submission, with excess records truncated. No explicit residue-length cutoff is displayed on the interface. No quantitative server-throughput benchmark is reported here.

Because the BTP640 benchmark peptides ranged from 2 to 39 amino acids in length, predictions for sequences far outside this range should be interpreted cautiously. Webserver outputs should be used for sequence-level screening and prioritization, not as direct sensory bitterness-intensity measurements in real food matrices.

S7. Figure 2 graphical summary

Figure 2A and 2B were prepared in GraphPad Prism from the grouped performance values obtained under the seven candidate classifiers. The bars denote medians. The graphical error bars in the originally submitted panels were not based on a prespecified calculation of a standard deviation, standard error, or confidence interval and have been removed from the revised figure. No inferential interval or formal hypothesis test is reported for these panels. Figure 2C is a descriptive boxplot: the central line is the median, the box is the interquartile range, and the whiskers extend from the minimum to the maximum.
